# Metformin suppresses calcium oxalate crystal-induced kidney injury by promoting Sirt1 and M2 macrophage-mediated anti-inflammatory activation

**DOI:** 10.1038/s41392-022-01232-3

**Published:** 2023-01-27

**Authors:** Haoran Liu, Chen Duan, Xiaoqi Yang, Jianhe Liu, Yaoliang Deng, Hans-Göran Tiselius, Zhangqun Ye, Tao Wang, Jinchun Xing, Hua Xu

**Affiliations:** 1 Department of Urology, The First Affiliated Hospital of Anhui Medical University, 230000 Hefei, China; 2 Bio-X, Stanford University, 94303 Stanford, California USA; 3 Department of Urology, Tongji Hospital, Tongji Medical College, Huazhong University of Science and Technology, 430000 Wuhan, China; 4 Department of Urology, The Second Affiliated Hospital of Kunming Medical University, 650000 Kunming, China; 5 Department of Urology, The First Affiliated Hospital of Guangxi Medical University, 530000 Nanning, China; 6 Division of Urology, Department of Clinical Sciences, Intervention and Technology, Karolinska Institutet, 13820 Stockholm, Sweden; 7 Department of Urology, The First Affiliated Hospital of Xiamen University, 361000 Xiamen, China; 8 Cancer Precision Diagnosis and Treatment and Translational Medicine Hubei Engineering Research Center, 430000 Wuhan, China; 9 Department of Biological Repositories, Zhongnan Hospital of Wuhan University, 430000 Wuhan, China; 10 Department of Urology, Zhongnan Hospital of Wuhan University, 430000 Wuhan, China

**Keywords:** Urology, Kidney diseases, Inflammation

**Dear Editor**,

Kidney stones are one of the most common clinical diseases in urology. Approximately 80% of renal calculi are composed of calcium oxalate (CaOx) crystals, which are gradually deposited in the medullary collecting duct or in the renal interstitium, causing nephrocalcinosis.^1^ Although CaOx nephrocalcinosis is usually asymptomatic, it can also induce severe inflammation and necrosis of renal tubular epithelial cells (TECs). Necrotic TECs release various damage-associated molecular patterns (DAMPs), which induce renal necroinflammation by activating Toll-like receptor 4 (TLR4) and secreting IL-1β, which triggers proinflammatory M1 macrophages (M1Mϕs) and then amplifies CaOx-induced renal injury,^2^ which can induce further crystal deposition and eventually lead to end-stage renal disease.^3^ However, information on the pharmacotherapeutics of kidney crystal formation remains limited. In addition, numerous studies have shown that CaOx crystal-induced oxidative stress and inflammatory injury to TECs are vital factors in the progression of crystalline nephropathy.^4^ Therefore, putting renal inflammation under control is expected to alleviate CaOx-induced inflammatory responses and inflammation-related tubular cell injury.

In principle, the inflammatory responses induced by CaOx crystals involve diverse downstream effectors. Therefore, targeting particular cytokines or even signaling pathways may not be sufficient to alleviate kidney injury. Metformin (Met) is a pleiotropic drug that functions mainly by targeting oxidative stress and inflammation. It has been reported to protect renal tubular cells from inflammation, apoptosis, reactive oxygen stress, and endoplasmic reticulum stress in acute or chronic kidney diseases.^5^ Sirtuin1 (Sirt1), an NAD^+^-dependent protein deacetylase, is significantly activated by Met-mediated promotion of NAD^+^ and the NAD^+^/NADH ratio and participates in various cellular processes, such as suppressing inflammation, enhancing M2Mϕ polarization, reducing oxidative stress and maintaining mitochondrial function. Determination of whether Met can systemically tune down exaggerated immune responses via inflammatory signaling cascades and can be applied as a therapeutic agent to treat CaOx-induced kidney injury is urgently needed.

In this study, we sought to understand the underlying pathways regulated by Sirt1 and explored the mechanism by which Met regulates CaOx nephrocalcinosis. We pretreated mice with increasing doses of Met (150, 200, and 250 mg/kg/d) for 3 days, after which a model of CaOx nephrocalcinosis was established for 7 days (along with Met treatment). Reduced CaOx crystal deposition was observed when the Met concentration increased on day 10. Importantly, Met attenuated kidney injury in TECs caused by CaOx nephrocalcinosis in a dose-dependent manner (Fig. 1a). Moreover, Sirt1 expression in Randall’s Plaque was lower than that in normal renal papillae in patients who underwent nephrectomy at Tongji Hospital (Supplementary Fig. S1a–d). Collectively, Met suppressed crystal deposition and kidney injury in the CaOx nephrocalcinosis mouse model, probably mediated by Sirt1.

**Fig. 1:**
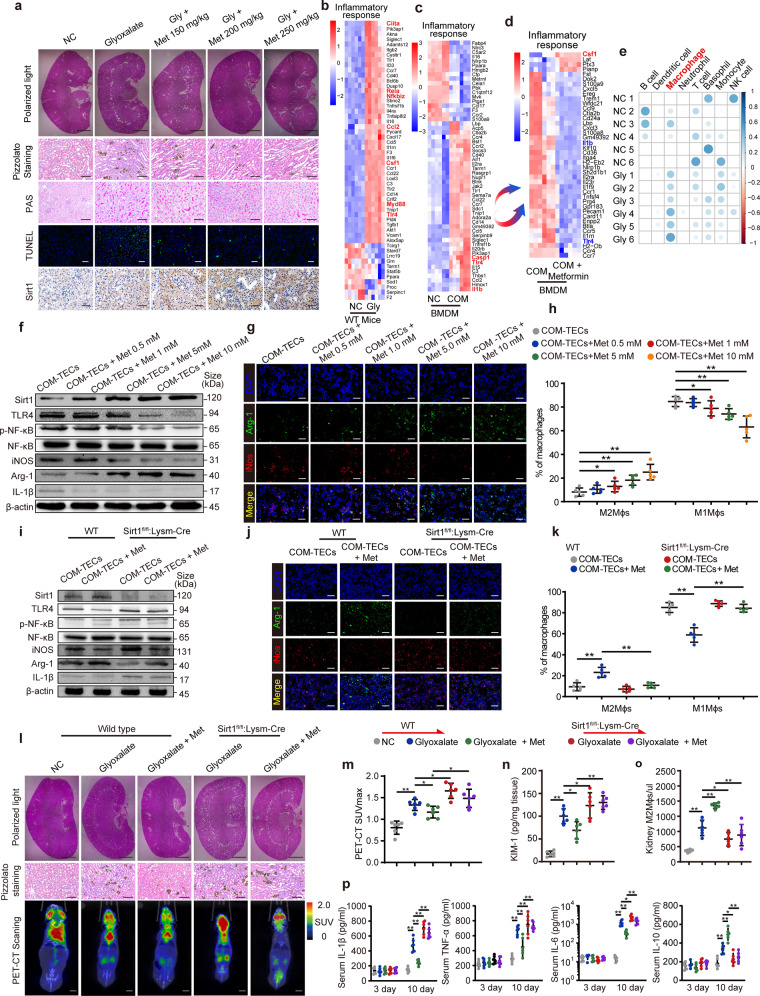
Met attenuates CaOx nephrocalcinosis-induced crystal deposition and kidney inflammatory injury by activating Sirt1 and M2Mϕs polarization. **a** The deposition of renal CaOx crystals in the corticomedullary junction of mice (*n* = 6) treated with increasing concentrations of Met was measured via polarized light optical microscopy (20×, scale bar: 2 mm). Pizzolato staining further showed CaOx crystal deposition (200×; scale bar: 80 μm). PAS staining (200×; scale bar: 80 μm) revealed tubular injury, TUNEL (200×; scale bar: 100 μm) staining revealed cell death and Sirt1 IHC staining (800×; scale bar: 20 μm) in kidney tissues. Heatmap cluster showing significantly altered mRNAs (|log2FC| ≥ 1.5, red, upregulated blue, downregulated) in the inflammatory response pathway in the WT-NC vs. WT-Gly mice (**b**), NC vs. COM in BMDMs (**c**) and COM vs. COM + Met in BMDMs (**d**). **e** Matrix dot plot enrichment analysis of the immune cell types that infiltrated the mouse kidney. In the BMDM-COM-stimulated TECs coculture system, BMDMs were treated with Met at increasing concentrations. **f**, **i** Expression of the indicated proteins in BMDMs detected by Western blots. β-actin served as a normal control. **g**, **j** Immunofluorescence was used to detect Arg-1 (green) and iNOS (red) distribution in BMDMs (800×; scale bar: 40 μm). **h**, **k** Flow cytometry analysis of the BMDM polarization state of M1ϕs and M2ϕs. WT and Sirt1^fl/fl^:Lysm-Cre mice were pretreated with Met, followed by intraperitoneal injection of glyoxylic acid. **l** Polarized light optical microscopy (20×, scale bar: 2 mm) and Pizzolato staining (200×; scale bar: 80 μm) showed CaOx crystal deposition in the renal corticomedullary junction. **m** ^18^F-FDG micro-PET-CT scanning was employed to assess the renal inflammation state in the mice with CaOx nephrocalcinosis (scale bar: 6 mm). **n** A renal injury marker (KIM-1) was used to evaluate renal inflammatory injury (*n* = 6). **o** Kidney-infiltrated M2Mϕs (CD45^+^MHCII^+^F4/80^+^CD11b^+^CD206^+^) were detected by flow cytometry. **p** Mouse serum IL-1β, TNF-α, IL-6, and IL-10 levels were detected by enzyme-linked immunosorbent assays. Data are the means ± SEMs of three independent experiments. *P < 0.05; **P < 0.01, based on Student’s t tests or one-way ANOVA (**h**, **k**, **m**–**p**)

To discover key functional gene sets that are potential therapeutic targets for Met in CaOx nephrocalcinosis, we performed transcriptional profiling by RNA sequencing (RNA-seq) on glyoxalate (Gly)- or saline-treated mouse kidneys (GSE192703). Gly-induced CaOx nephrocalcinosis caused the upregulation of 2596 genes and the downregulation of 2161 genes (|log_2_FC |≥ 1.5; *P* value < 0.05). Gene Ontology enrichment and differential gene expression confirmed that the presence of CaOx crystals increased a spectrum of proinflammatory responses (NF-κB, RELA, Myd88 and TLR4), macrophages recruitment and polarization (Ciita, Csf1, Ccl2, TLR4) (Fig. 1b and Supplementary Fig. S2a–d). To further determine which type of immune cell infiltration plays a crucial role in the CaOx-induced immune response, we used significant genes in our bulk RNA-seq profile to identify matching cell-type signatures in the Cellkb Immune database (https://www.cellkb.com/immune). The matrix dot plot enrichment of eight immune cell types showed a significant increase in renal macrophages infiltration in the Gly group (Fig. 1e), indicating that this factor may be involved in the inflammatory injury response associated with CaOx nephrocalcinosis.

Given the anti-inflammatory effect of Met and its known regulatory effects on macrophages, we next performed RNA-seq on calcium oxalate monohydrate (COM)-stimulated bone marrow-derived macrophages (BMDMs) (GSE192593) pretreated with Met or PBS in TECs or COM-TECs (NC vs. COM, COM vs. COM + Met). Analysis of differential gene expression confirmed that COM-TECs led to upregulation of a spectrum of proinflammatory transcripts in BMDMs (Fig. 1c). KEGG pathway enrichment analysis showed that the “NF-κB pathway, Toll-like receptor signaling and cytokine receptor interaction” were ranked in the top 20 significant KEGG pathways in the COM-TECs-stimulated BMDMs group (Supplementary Fig. S3a, b), which was consistent with the activated inflammatory response in the mice with Gly-induced CaOx nephrocalcinosis. However, the expression of this inflammatory response-related gene (TLR4, IL-1β, Ccr and Cxcl family) was significantly reversed after Met treatment (Fig. 1d and Supplementary Fig. S3c–e). To determine the effects of Met on these proinflammatory genes in vivo, we performed IHC staining, which revealed dramatically increased Met-dependent Sirt1 staining and decreased TLR4 and IL-1β staining (Supplementary Fig. S4a). In addition, qRT–PCR revealed a significant decrease in TLR4, IL-1β, and iNOS expression, whereas Sirt1 and Arg-1 expression was enhanced in the kidneys of the Met-treated mice with CaOx nephrocalcinosis (Supplementary Fig. S4b, c). Moreover, Pearson’s correlation coefficient analysis indicated a negative correlation between the expression of Sirt1 and the expression of TLR4 and IL-1β (Supplementary Fig. S4d, e). These data suggest that Met may abrogate CaOx-induced inflammatory injury through Sirt1.

We hypothesized that Met exerts its anti-inflammatory effects partly by interfering with TLR4 and IL-1β through Sirt1 in macrophages and modulating macrophages polarization. To evaluate this hypothesis, we cocultured COM-stimulated TECs with Met-treated BMDMs. Fig. 1f and Supplementary Fig. S5a, b show that Met treatment activated Sirt1 expression, which subsequently downregulated TLR4, p-NF-κB, IL-1β and iNOS (M1Mϕs markers) and upregulated Arg1 (an M2Mϕs marker). Moreover, Fig. 1g and Supplementary Fig. S5c show that Met increased M2Mϕs polarization while inhibiting M1Mϕs polarization. In addition, flow cytometry showed that Met significantly reversed the promotion of M1Mϕs polarization after COM treatment in a Met dose-dependent manner (Fig. 1h). To demonstrate the vital role of Sirt1 in Mϕs polarization, we cocultured Sirt1cKO BMDMs from Sirt1^fl/fl^:Lysm-Cre mice and COM-TECs from WT mice. As expected, Sirt1 knockout (KO) significantly promoted M1Mϕs polarization and increased TLR4 signaling (Fig. 1i and Supplementary Fig. S5d, e) Moreover, loss of Sirt1 reversed the effect of Met in promoting M2Mϕs polarization (Fig. 1j, k and Supplementary Fig. S5f). The results described above strongly indicated that Met treatment suppressed the CaOx-triggered inflammatory responses by activating Sirt1 in vitro.

We further explored whether the effects of Met on CaOx-induced renal inflammation and crystal deposition are Sirt1dependent. We pretreated the WT and Sirt1^fl/fl^:Lysm-Cre mice with Met for 3 days and developed a CaOx nephrocalcinosis mouse model by the intraperitoneal injection of glyoxylic acid. We determined that Met significantly reduced kidney CaOx crystal deposition in the WT mice, while Sirt1cKO reversed this effect (Fig. 1l and Supplementary Fig. S6a, b). We analyzed ^18^F-FDG uptake by micro-PET-CT to determine the local renal inflammatory response. Micro-PET-CT imaging revealed that Met decreased ^18^F-FDG uptake in the WT mouse kidneys, while these parameters were enhanced in the Sirt1^fl/fl^:Lysm-Cre mice (Fig. 1l, m). Furthermore, the levels of mouse serum inflammatory markers (IL-1β, TNF-α, IL-6, IL-10), serum creatinine, and blood urea nitrogen (BUN) were determined to evaluate the systemic inflammatory status and renal function, which demonstrated the same effects (Fig. 1p and Supplementary Fig. S6c, d). PAS, TUNEL, DHE and oxidative marker (SOD2 and HO-1) staining further confirmed that Sirt1cKO reduced the protective effects of Met on CaOx crystal-induced renal injury (Supplementary Fig. S7a–f). In addition, we demonstrated that Met alleviated renal tubular injury, increased the renal infiltration of M2Mϕs populations, and suppressed TLR4 inflammatory signaling in a Sirt1-dependent manner (Fig. 1n, o and Supplementary Fig. S8a–e). In summary, Met depends on Sirt1 to exert its anti-inflammatory and renal protective effects by regulating Mϕs polarization.

In conclusion, the results demonstrated that Met can attenuate crystal deposition and kidney injury in a CaOx nephrocalcinosis mouse model by promoting Sirt1 activation and M2Mϕs polarization. A combination of Met with certain anti-oxalate drugs may provide a more effective treatment and prevent recurrence in CaOx nephrocalcinosis. Our study provides strong evidence supporting the therapeutic and preventative potential of using Met in clinical settings.

## Supplementary information

Supplemental Material

## Data Availability

All data needed to evaluate the conclusions in the paper are presented in the paper or the Supplementary Materials. Any data associated with this study are available from the corresponding author upon reasonable request. The RNA-seq data in this study can be accessed in GEO dataSets GSE192703 and GSE192593.
