# Supplementary material for: Metformin suppresses calcium oxalate crystal-induced kidney injury by promoting Sirt1 and M2 macrophage-mediated anti-inflammatory activation
Source: Signal Transduct Target Ther. 2023 Jan 27;8:38. doi: 10.1038/s41392-022-01232-3 (PMC9879973; doi:10.1038/s41392-022-01232-3)
Supplement: Supplementary file 1 — Supplemental Material [file 41392_2022_1232_MOESM1_ESM.docx]

**Supplementary Materials for**

**Metformin suppresses calcium oxalate crystal-induced kidney injury by promoting Sirt1 and M2 macrophage-mediated anti-inflammatory activation**

Haoran Liu^1, 2^†, Chen Duan^3^†, Xiaoqi Yang^3^, Jianhe Liu^4^, Yaoliang Deng^5^, Hans-Göran Tiselius^6^, Zhangqun Ye^3^, Tao Wang^7^, Jinchun Xing^7^, Hua Xu^8, 9, 10^ *

†Haoran Liu and Chen Duan contributed equally to this work.

* Correspondence to: Hua Xu. Email: [xu-hua@whu.edu.cn](mailto:xu-hua@whu.edu.cn)

**This PDF file includes:**

**Materials and Methods**

**Figures S1-S9**

**Table S1**

**Materials and Methods**

**Animal Studies**

The animals used in the experiment were male C57BL/6J mice, 6-8 weeks old, purchased from the Hubei Experimental Animal Research Center. We obtained myeloid-specific Sirt1 knockout mice by hybridizing mice that carried a floxed Sirt1 allele (Sirt1^flox/flox^, T006657, GemPharmatech Inc., Nang Jing, China) and Lysm-cre transgenic mice (T003822, GemPharmatech Inc., Nang Jing, China). Sirt1 conditional knockout in bone marrow-derived macrophages (BMDMs) was confirmed at both the DNA and protein levels (Supplementary Fig. S9a-d). Animals were raised in the specific-pathogen-free animal facility of Tongji Hospital in compliance with the NIH Guide for the Care and Use of Laboratory Animals. To establish a mouse model of CaOx nephrocalcinosis, mice were intraperitoneally injected with normal saline or glyoxylate acid (Gly) (75 mg/kg/d, 200 μl) from day 4 to day 10. Mice in the treatment groups were intraperitoneally injected with 200 μl of Met at concentrations of 150, 200 and 250 mg/kg/d from day 1 to day 10. Animals were euthanized after 10 days, and kidney samples were collected at the time of euthanasia and fixed. The study (TJ 20180622-3) was approved by the Ethics Committee of Tongji Hospital, Tongji Medical College, Huazhong University of Science and Technology.

**Cell Culture**

BMDMs were isolated from myeloid-specific Sirt1 knockout (Sirt1^fl/fl^:Lysm-Cre) mice and wild-type (WT) mice. Tibias and femurs were removed from the dead mice, and the bone marrow was rinsed with cold sterile phosphate-buffered saline (PBS) and cultured in RPMI 1640 medium with 10% FBS and 50 ng/ml M-CSF for 7 days at 37°C and 5% CO_2_. After 5 days of culturing, the medium was replaced to remove unattached cells, and the adherent cells were further cultured for subsequent experiments. TECs were isolated and cultured from kidney tissue samples obtained from WT mice. First, the renal cortex was broken and digested with type-I collagenase for 30 min at 37°C, after which TECs were isolated by Percoll centrifugation. Immunostaining analysis with Hoechst dye and cytokeratin-18 was performed to determine the purity of TECs. To further study the effects of COM-stimulated TECs on Mϕs, we developed a BMDM-COM-stimulated TECs coculture system. BMDMs were plated in the upper chamber of 6-well Transwell plates with a pore size of 0.4 μm (Corning, USA), while TECs were plated in the lower chamber. In the coculture experiment with BMDMs and TECs, TECs were treated with COM (100 μg/mL) for 24 h.

**Positron Emission Tomography-Computed Tomography (PET-CT) Imaging**

^18^F-Fluorodeoxyglucose (^18^F-FDG) is a deoxyglucose analog that accumulates at high concentrations in tissues that utilize large amounts of glucose. Activated inflammatory cells that consume large amounts of glucose similarly exhibit high ^18^F-FDG uptake. Each animal received a caudal vein injection of 200 ± 10 μCi ^18^F-FDG. One hour later, each mouse was anesthetized with 2% isoflurane, after which a static PET scan was conducted for 10 min using a Trans-PET BioCaliburn 700 System (Raycan Technology Co., Ltd., Suzhou, China). The 3D-ordered subset expectation-maximization method (voxel size: 0.5×0.5×0.5 mm^3^) was used to reconstruct the data.

**Observation of the Deposition of Kidney CaOx Crystals**

Renal tissue was cut into 4 μm thick sections and stained with hematoxylin-eosin (HE). Polarized light optical microscopy (Zeiss, Oberkochen, Germany) was used to analyze the staining results. In addition, Pizzolato staining was performed to visualize kidney crystal deposition. CaOx crystal deposition in kidney sections was quantified with ImageJ software.

**Assessment of Renal Tubular Injury**

The renal tissue sections were stained using periodic acid Schiff (PAS) reagent to evaluate the following signs of renal tubular injury: sloughing of TECs, tubular atrophy, tubular dilation, tubular cast formation, and thickening of the tubular basement membrane. The grade of tubular injury was scored by calculating the percentage of impaired tubules (scoring: none, 0; < 25%, 1; 25–50%, 2; 51–75%, 3; and > 75%, 4). Ten consecutive and nonoverlapping fields in each tissue section were assessed at a magnification of 200×. Renal cell death was assessed by using the Cell Detection Kit (Absin, China) according to the manufacturers’ protocol. Ten magnified fields were randomly selected from each section for TUNEL staining to determine the positive cell count.

**Immunohistochemical (IHC) Staining**

Formalin was used for the fixation of renal specimens, and paraffin-embedded sections were made and stained with HE. For IHC staining, the primary antibodies were as follows: anti-Sirt1 (1:800, Abcam, USA), anti-TLR4 (1:1000, Abcam, USA), anti-SOD2 (1:1000, Abcam, USA), anti-NOX2 (1:1200, Abcam, USA), and anti-IL-1β (1:500, Absin, USA). Images were obtained with a Leica SCN400 scanner. The relative expression of each gene of interest was analyzed using ImageJ software.

**ELISA Experiments**

The levels of cytokines in the supernatants of cocultured systems and mouse serum were detected by ELISA kits. Kits for IL-1β (DY401), TNF-α (DY410), and IL-10 (DY417) were obtained from R&D (USA), and IL-6 (BMS603-2) was obtained from Thermo Fisher (USA). All kits were used in accordance with the manufacturers’ protocols.

**Quantitative PCR (qPCR)**

Total RNA was extracted from BMDMs by TRIzol Reagent (Absin, China). The PrimeScript RT Reagent Kit (TaKaRa, Japan) was used to reverse transcribe RNA into complementary DNA. Finally, SYBR Green Master Mix (Yeasen, China) was used for qPCR experiments following the manufacturers’ instructions. We used β-actin as a reference gene. The primer sequences are shown in Supplementary Table S1.

**Western blotting**

RIPA lysis buffer was used to extract total protein from BMDMs, and the protein concentration was measured with the BCA Protein Assay Kit (Beyotime Biotech, China). Extracted protein containing loading buffer was subjected to electrophoresis in a 10% SDS-polyacrylamide gel and incubated at 4°C overnight with primary antibodies against Sirt1 (8496S, 120 kDa, 1:1,000, CST, USA), iNOS (GB13495, 131 kDa, 1:800, Servicebio, China), TLR4 (GB11519, 95 kDa, 1:12000, Servicebio, China), NF-κB p65 (BA0610, 65 kDa, 1:1000, Boster, China), Arginase 1 (BA3796-2, 40 kDa, 1:2,000, Boster, China), IL-1β (3553, 17 kDa, 1:1,000, CST, USA), and β-actin (GB11001, 43 kDa, 1:2,000, Servicebio, China). Afterward, the membrane strips were incubated with HRP-labeled secondary antibody for 1 h at 37°C and visualized with LumiBlue (TM) ECL Express (Novus). The average relative densities of the proteins were quantified with ImageJ software after normalization to β-actin. All experiments were independently replicated three times.

**Immunofluorescence**

BMDMs were fixed and blocked with 4% paraformaldehyde and 5% goat serum. When these steps were completed, we added Arg1 antibody (ab239731, 1:600, Abcam, USA) or iNOS antibody (ab210823, 1:400, Abcam, USA) and incubated overnight in a refrigerator at 4°C. This was followed by incubation with Alexa Fluor 488- or CY3-conjugated secondary antibodies for 1 h at 37°C. Subsequently, DAPI was added for 5 min for restaining, and the cells were studied with fluorescence microscopy (Nikon, Japan).

**Renal Macrophage Isolation**

After perfusion with 4°C PBS to remove the blood, mouse kidneys were excised, decapsulated, minced, and digested with collagenase and DNase. Digested tissue was passed through a cell strainer with a 40-μm mesh size. The filtrate was then washed, incubated with anti-CD16/32 and stained with anti-CD45, anti-CD11b, anti-MHCII, anti-F4/80, and anti-CD206. After incubation, the cells were washed with FACS buffer, followed by analysis in a flow cytometer. Counting beads (PCB100; Thermo Fisher Scientific, Langenselbold, Germany) were used to determine the number of cells.

**Flow Cytometry**

The polarization state of BMDMs was observed with F4/80 (PE, BD Pharmingen™, 565410), CD11b (FITC, BD Pharmingen™, 557396), CD11c (PE-Cy7, BD Pharmingen™, 558079), and CD206 (Alexa Fluor 647, BD Pharmingen™, 565250) antibodies at 37°C. All results were analyzed by FC (BD Biosciences, USA) and FlowJo V10 (Tree Star, CA).

**Measurement of ROS Generation**

Kidney tissue ROS was determined by dihydroethidium (DHE) staining. For this experiment, kidney tissues were frozen and embedded in optimum cutting temperature (O.C.T.) matrix (SAKURA Tissue-Tek). Frozen renal sections were incubated with DHE at 37°C for 30 min, after which excess dye was removed by three washes with PBS. The images were observed under fluorescence microscopy (Nikon, Japan) as previously described.

**Malondialdehyde (MDA), Kidney Injury Molecule-1 (KIM-1) and Glutathione (GSH) Levels**

The levels of MDA and KIM-1, which were used to assess cell membrane destruction and lipid peroxidation, respectively, in the supernatants of cocultured RTCs were measured by using commercial MDA and KIM-1 assay kits (Beyotime Biotech, China). To further detect and assess antioxidative enzyme activities and GSH levels in RTCs, we used commercially available assay kits (Beyotime Biotech, China).

**Determination of Serum Creatinine and Blood Urea Nitrogen (BUN) Levels**

Serum was collected from mice in the experimental groups on days 3 and 10, and serum BUN and creatinine levels were measured using commercial kits (Stanbio Laboratory, USA).

**Plasma Glucose Measurement**

After 10 days of Met treatment as previously described, mice were fasted for 16 h, and blood was collected from the tail vein. Blood glucose levels were determined using a glucose meter (Glucotrend II; Roche Diagnostics). Mouse blood glucose levels decreased with increasing metformin dosage (Supplementary Fig. S2a).

**Statistical Analysis**

GraphPad Prism 8.0 was used to analyze the data. The experimental measurement data are expressed as the mean ± standard deviation (M ± SEM). Student’s t test and one-way analysis of variance (ANOVA) were used to analyze the differences between experimental groups. Pearson’s correlation analysis was applied to analyze the correlations between different genes. A *p* value < 0.05 indicated statistical significance.

**Supplementary Fig. S1.**

**
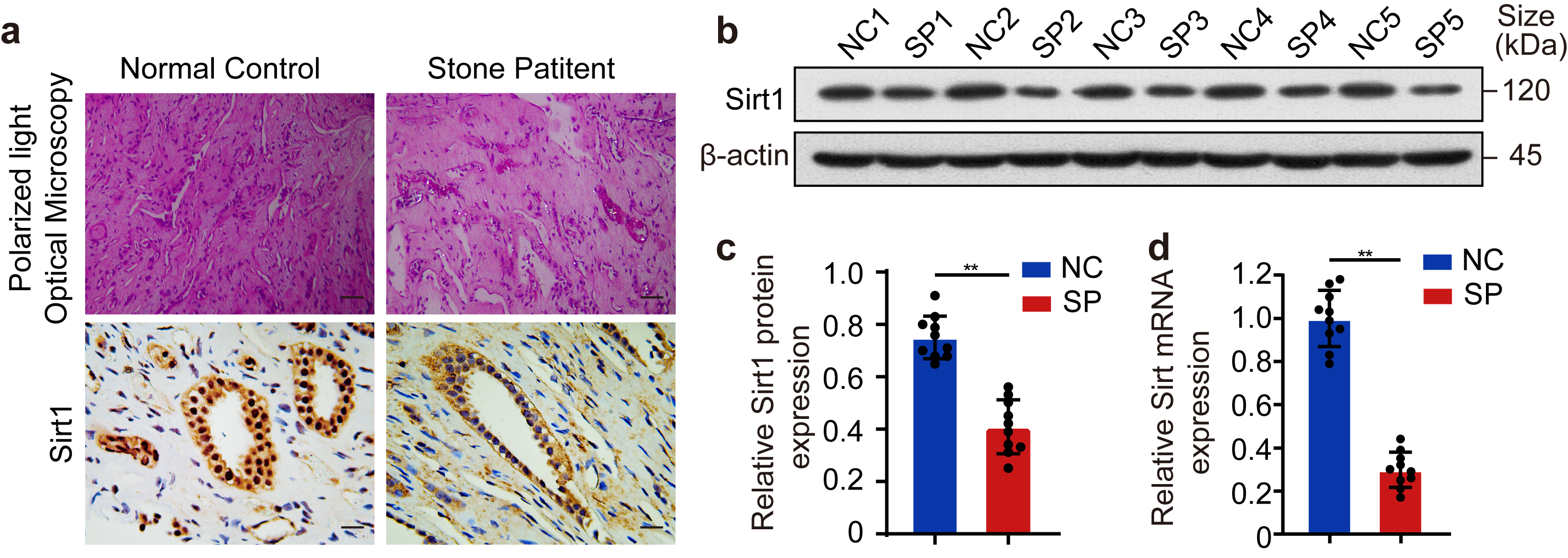
**

**Fig. S1. Reduced Sirt1 expression in Randall’s plague from kidney stone patients. a** Renal papillae sections containing Randall’s plaques and normal renal papillae sections were visualized via polarized light optical microscopy (scale bar: 50 μm) and IHC staining of renal papillae Sirt1 (scale bar: 10 μm). Sirt1 protein and relative mRNA expression in kidney stone patients and normal controls was detected by Western blot **(b, c)** and real-time quantitative PCR (qPCR) analysis **(d)**. β-Actin served as an internal control. One representative plot of n = 5 patients is shown. *P < 0.05; **P < 0.01, as assessed via Student’s t test (c).

**Supplementary Fig. S2.**

**
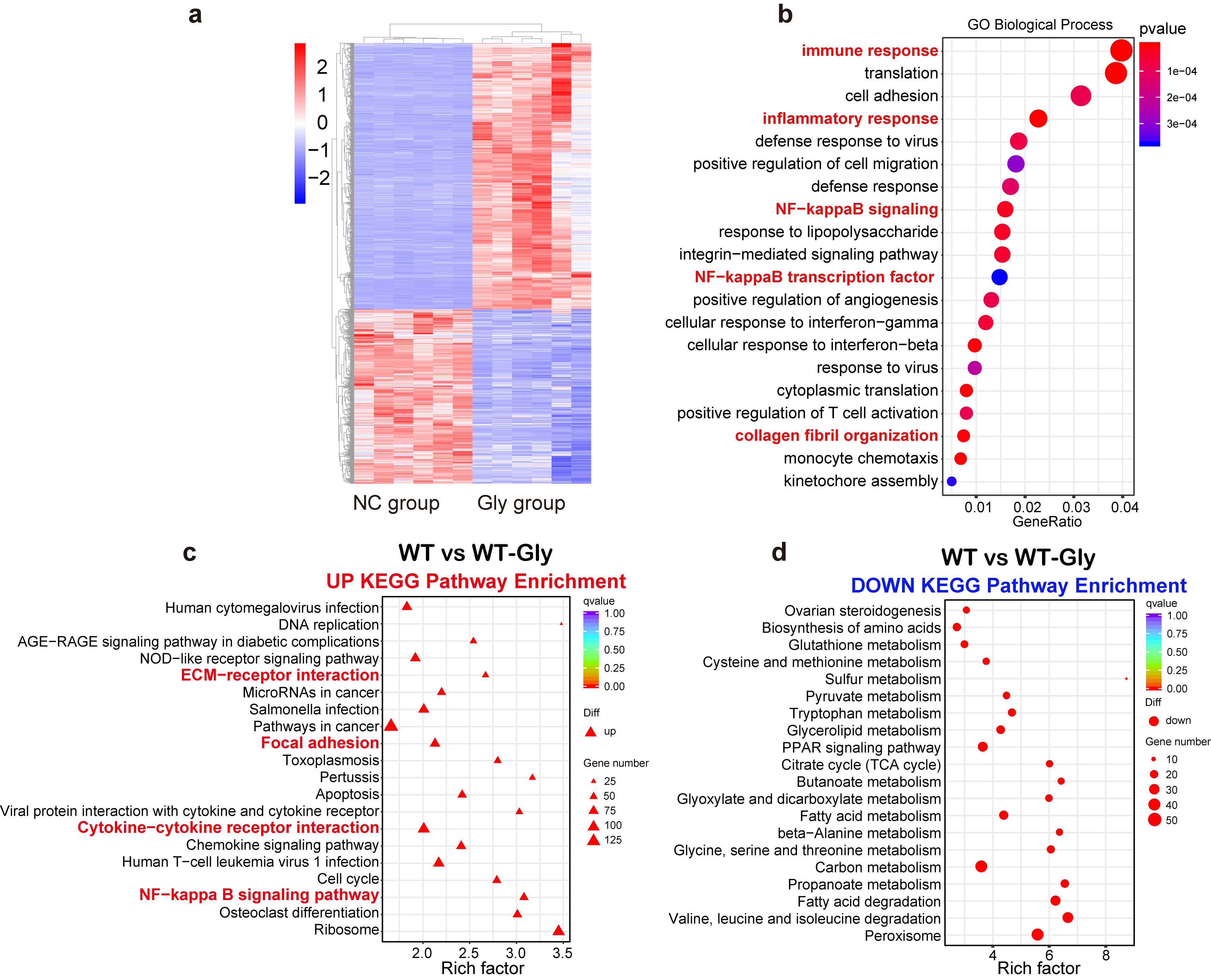
**

**Fig. S2. RNA-seq analysis in kidney across comparison of WT-NC vs. WT-Gly mice. a** RNA-seq heatmap showing significantly altered mRNAs in the kidneys of glyoxylate-induced CaOx nephrocalcinosis mouse models. **b** Gene Ontology analysis of biological processes showed that the immune response, the inflammatory response and NF-κB signaling activation play vital roles in CaOx nephrocalcinosis. **c, d** Upregulated and downregulated KEGG pathway analysis in the kidneys of WT-NC vs. WT-Gly mice.

**Supplementary Fig. S3.**

**
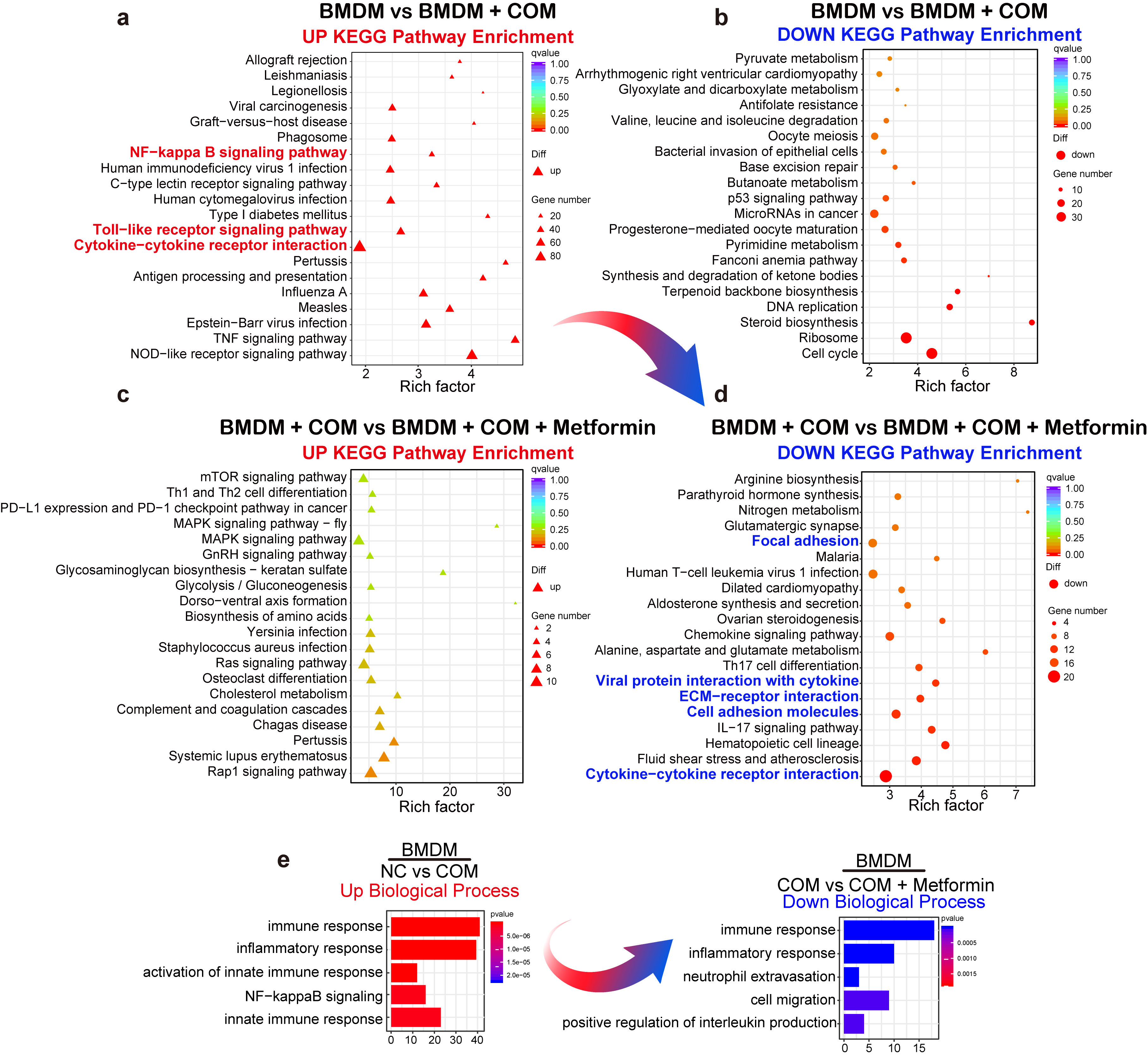
**

**Fig. S3. Met treatment significantly reversed the COM-induced inflammatory response in macrophages.** Gene Ontology analysis showed changes in biological processes after Met treatment in COM-TECs-stimulated BMDMs. Upregulated KEGG pathways in the kidneys of NC vs. COM **(a)** and COM vs. COM + Met in BMDMs **(c)**. Downregulated KEGG pathways in the kidneys of NC vs. COM **(b)** and COM vs. COM + Met in BMDMs **(d)**. **e** Gene Ontology analysis showed changes in biological processes after Met treatment in COM-TECs-stimulated BMDMs.

**Supplementary Fig. S4.**

**
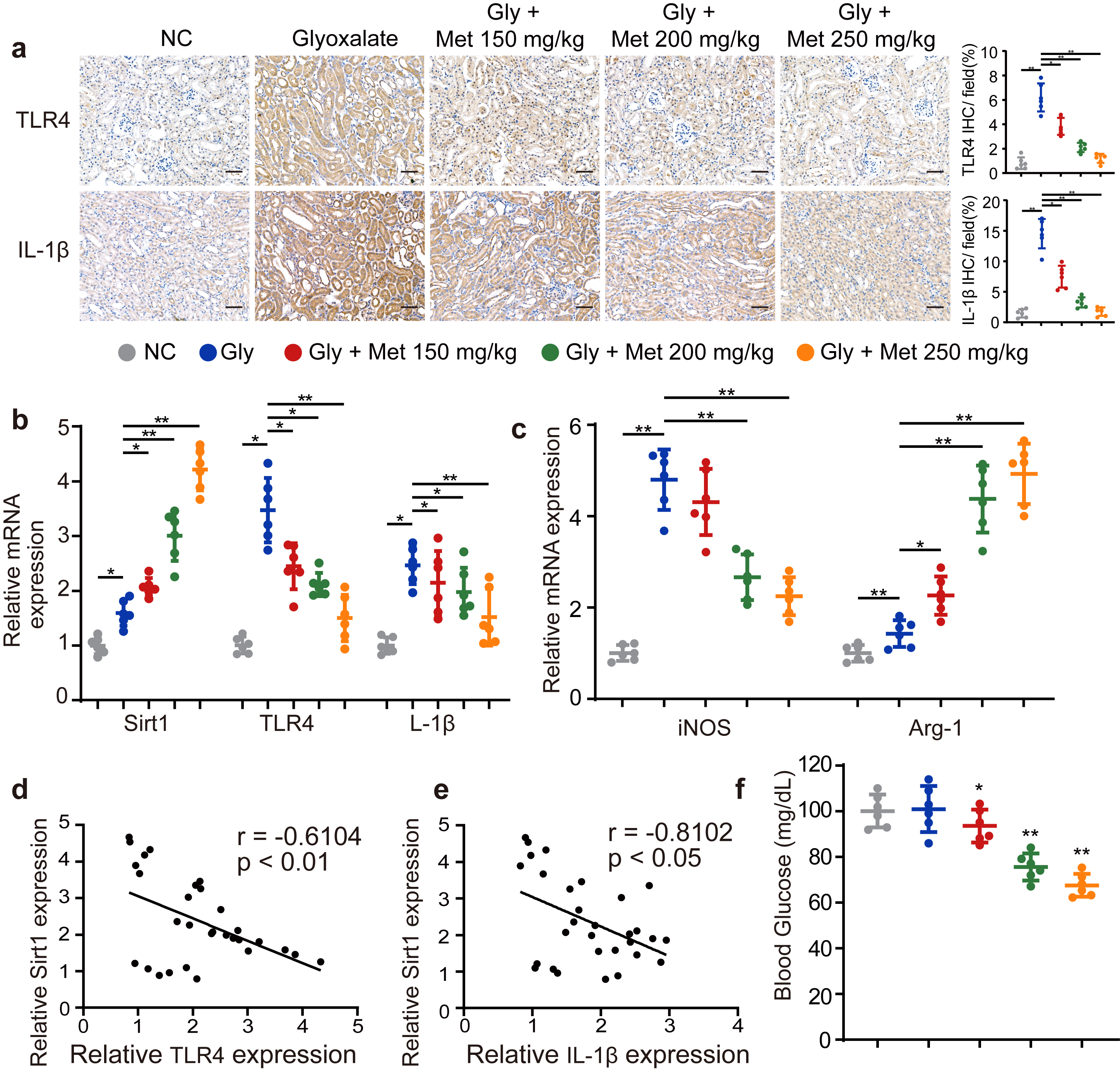
**

**Fig. S4.** **Sirt1 activation suppressed TLR4 and IL-1β and attenuated M1Mϕs polarization markers in vivo. a** IHC staining for TLR4, and IL-1β in the kidneys of Met-treated mice with CaOx nephrocalcinosis (200×; scale bar: 40 μm). The ratio of areas with IHC-positive expression of kidney Sirt1, TLR4, and IL-1β in the CaOx nephrocalcinosis mouse model (n = 6) treated with Met at different concentrations. **b, c** Real-time quantitative PCR (qPCR) analysis of Sirt1, TLR4, IL-1β, iNOS and Arg-1 expression in the CaOx nephrocalcinosis model. β-Actin was used as the internal control. **d, e** Pearson’s correlation analysis clarified the relationship between Sirt1 expression and the expression of TLR4 or IL-1β. **f** Fasting blood glucose in mice after Met treatment at different dosages. The data are shown as the mean ± SD. One representative plot of n = 6 mice is shown. *P < 0.05; **P < 0.01, as assessed via Student’s t test (f) or one-way ANOVA (a-c).

**Supplementary Fig. S5.**

**
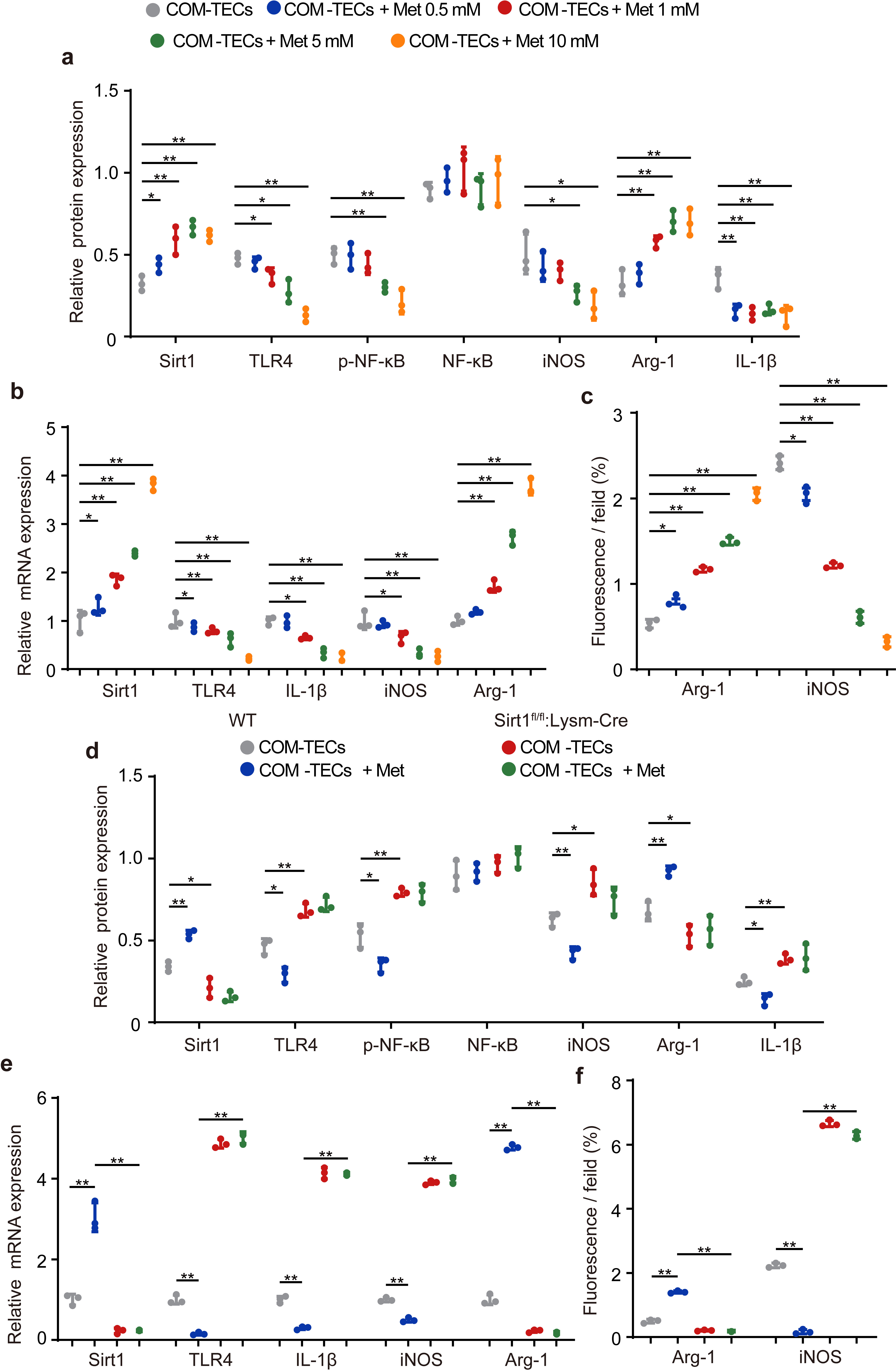
**

**Fig. S5**. **Met protected TECs from the COM-triggered inflammatory response via Sirt1.** **a, b** Western blot quantification and qPCR analysis of Sirt1, TLR4, IL-1β, iNOS, and Arg-1 expression in BMDMs cocultured with COM-TECs in the absence or presence of different concentrations of Met. Protein expression are relative to β-actin expression. β-Actin was used as the internal control. **c** IF analysis of BMDM polarization with anti-iNOS and Arg-1 for Fig. 1g. **d, e** Western blot quantification and qPCR analysis of Sirt1, TLR4, IL-1β, iNOS, and Arg-1 expression in WT or Sirt1 KO BMDMs cocultured with COM-TECs. β-Actin was used as the internal control. **f** IF analysis of BMDM polarization with anti-iNOS and Arg-1 antibodies in Fig. 1j. *P < 0.05; **P < 0.01, as determined by one-way ANOVA (a-f).

**Supplementary Fig. S6.**


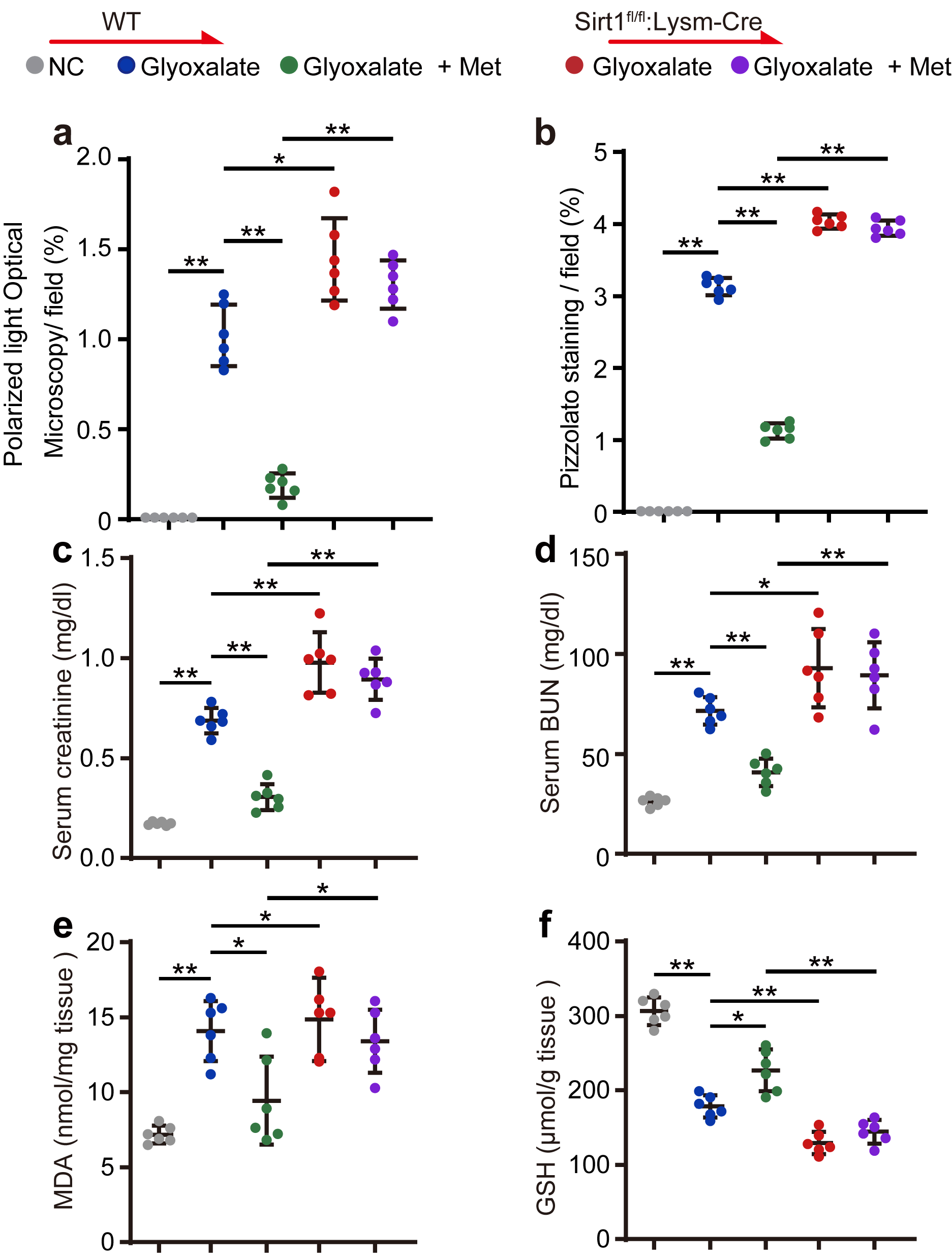


**Fig. S6.** **Loss of Sirt1 abolished the effect of Met on** **CaOx crystal deposition in vivo.** **a** The ratio of the areas of kidneys with crystal deposition, as detected by polarized light optical microscopy. **b** The ratio of the areas of kidneys with crystal deposition in Pizzolato staining. **c, d** Serum creatinine and BUN were used to assess renal function (n = 6). **e, f** Antioxidant (GSH) and injury markers (MDA) were used to evaluate renal oxidative injury (n = 6). The data are shown as the mean±SD. One representative plot of n = 6 mice is shown. *P < 0.05; **P < 0.01, as determined by one-way ANOVA (a-f).

**Supplementary Fig. S7.**

**
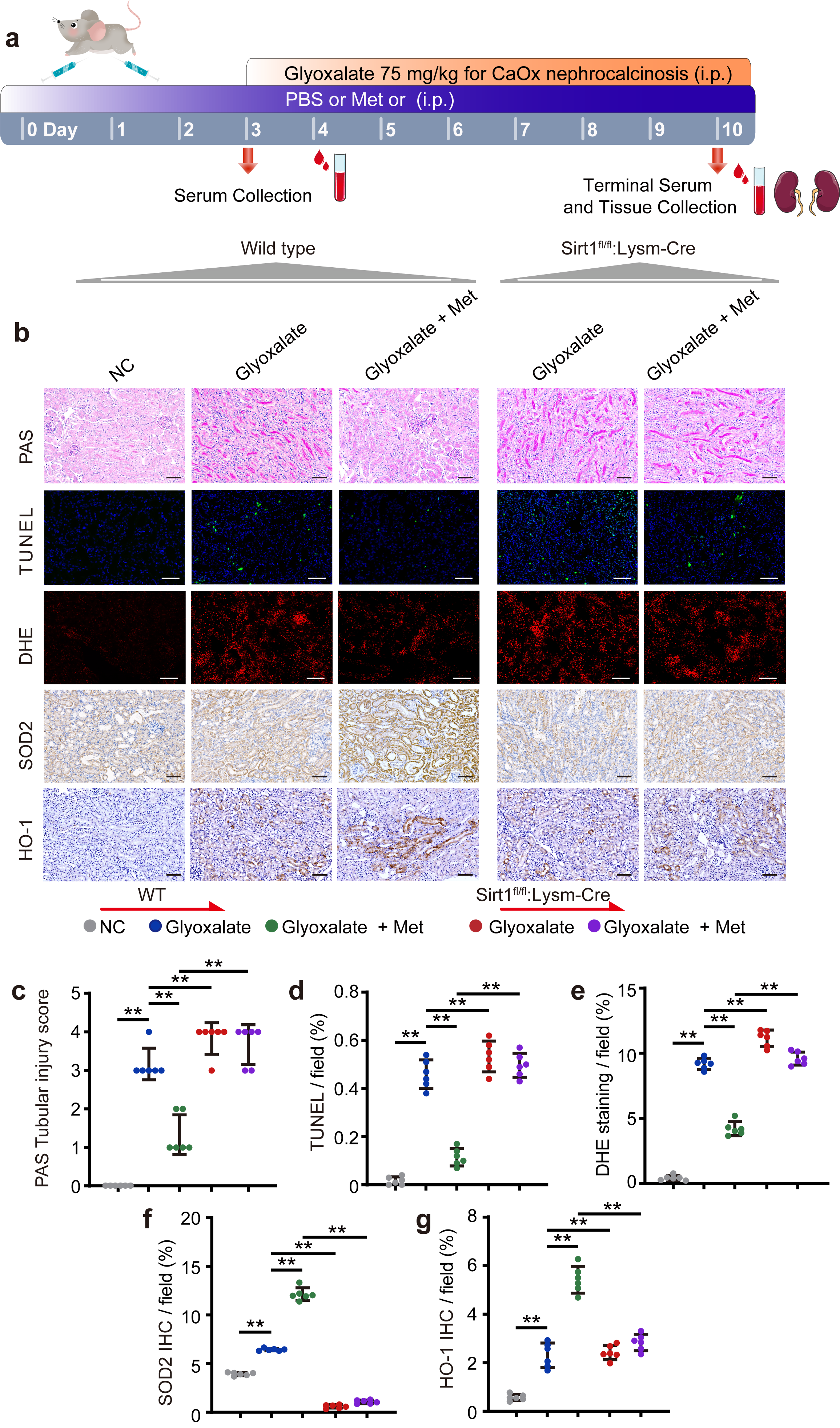
**

**Fig. S7. Loss of Sirt1 abolished the effect of Met on nephrocalcinosis-induced inflammatory kidney injury.** **a** Mice in the treatment groups were intraperitoneally injected with 200 μl of Met at concentrations of 250 mg/kg/d from day 1 to day 10. To establish a mouse model of CaOx nephrocalcinosis, mice were intraperitoneally injected with normal saline or glyoxylate acid (Gly) (75 mg/kg/d, 200 μl) from day 4 to day 10. Blood samples were collected and analyzed on day 3 and day 10. Mice were euthanized after 10 days, and kidney samples were collected at the time of euthanasia and fixed. **b** PAS staining was performed to detect tubular damage (200×; scale bar: 40 μm). TUNEL staining revealed cell death in kidney tissues (200×; scale bar: 50 μm). DHE staining was used to measure ROS generation in mouse renal tissue. (200×; scale bar: 50 μm). IHC staining was used to measure SOD2 and HO-1 in renal tissue (200×; scale bar: 40 μm). **c** The tubular injury score was determined by PAS staining. **d** The average number of TUNEL-positive cells per high-power field (200×; n=10 fields per section). **e** The ratio of the areas with positive cells observed in DHE staining. **f, g** The ratios of the areas with positive expression of SOD2 and HO-1, as determined by IHC. The data are shown as the mean±SD. One representative plot of n = 6 mice is shown. *P < 0.05; **P < 0.01, as determined by one-way ANOVA (c-g).

**Supplementary Fig. S8.**


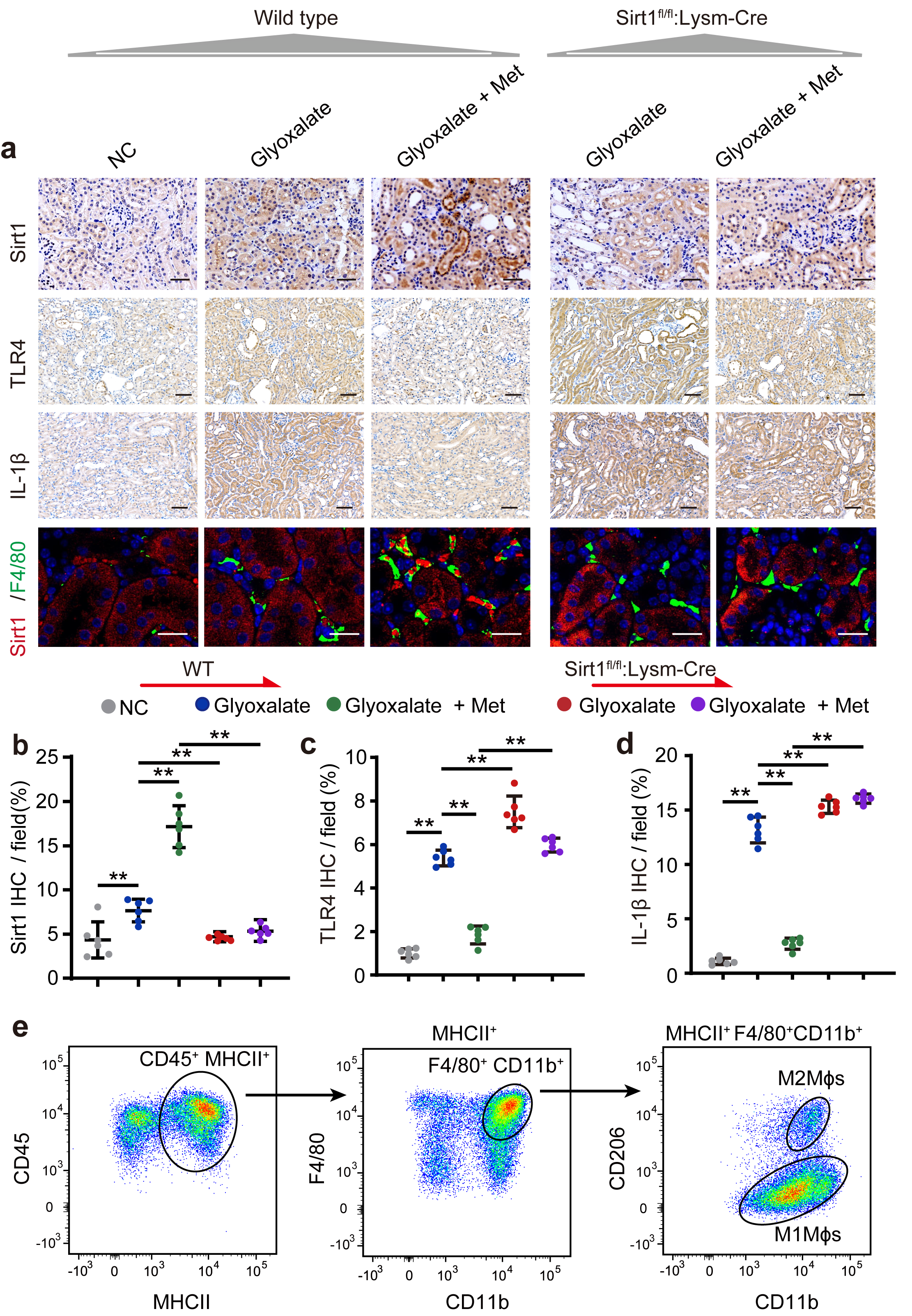


**Fig. S8. Loss of Sirt1 abolished the effect of Met on nephrocalcinosis-induced inflammatory activation and macrophage polarization. a** IHC staining was used to measure Sirt1 (800×; scale bar: 20 μm), TLR4, and IL-1β in renal tissue (200×; scale bar: 40 μm). Sirt1 (red) and iNOS (green) showing the M1Mϕs distribution in mouse kidneys were detected by immunofluorescence (1000×; scale bar: 20 μm). **b, c, d** The ratios of the areas with positive expression of Sirt1, TLR4, and IL-1β, as determined by IHC. **e** Gating strategy to identify macrophages (CD45^+^MHCII^+^CD11b^+^F4/80^+^), proinflammatory M1-like macrophages (CD45^+^MHCII^+^F4/80^+^CD11b^+^CD206^-^), and alternatively activated M2-like macrophages (CD45^+^MHCII^+^F4/80^+^CD11b^+^CD206^+^).

**Supplementary Fig. S9.**

**
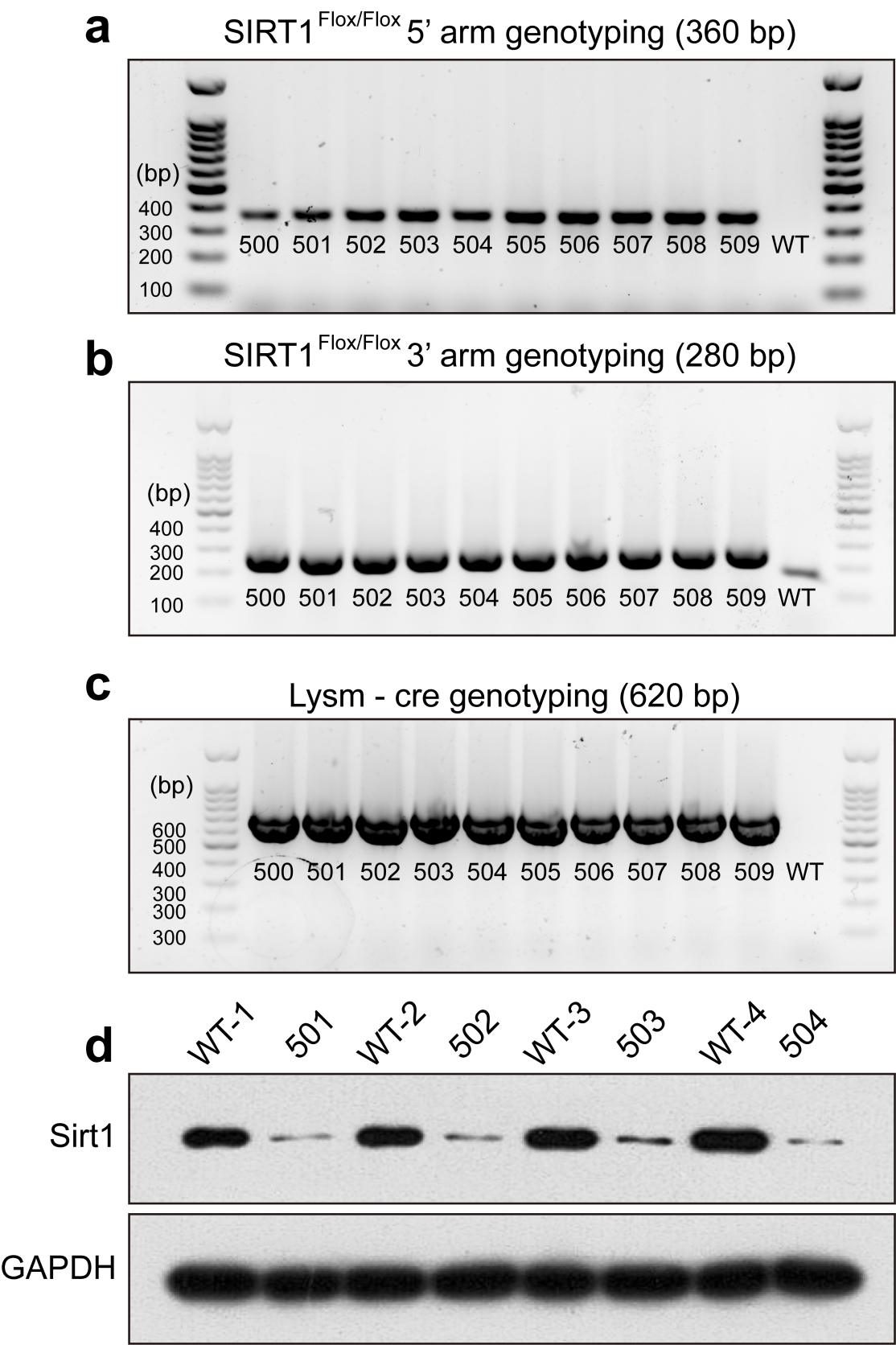
**

**Fig. S9. Genotype and Sirt1 protein in Sirt1^fl/fl^:Lysm-Cre mice.** **a, b** Gel showing the genotype for the 5’ and 3’ arms of the Sirt1^fl/fl^ gene (360 and 280 bp). **c.** Gel showing the genotype for Lysm-cre. **d** Western blot showing Sirt1 expression in both Sirt1^fl/fl^:Lysm-Cre and WT mice.

**Table S1.** List of primer sequences used for real-time qPCR analysis

| **Primer** | **Name** | **(5'-3')** | **SEQUENCE (5'-3')** |
| --- | --- | --- | --- |
| qRT–PCR | Sirt1 | Forward | TGATTGGCACCGATCCTCG |
|  |  | Reverse | CCACAGCGTCATATCATCCAG |
|  | TLR4 | Forward | TCTGGGGAGGCACATCTTCT |
|  |  | Reverse | AGGTCCAAGTTGCCGTTTCT |
|  | iNOS | Forward | CACCTTGGAGTTCACCCAGT |
|  |  | Reverse | ACCACTCGTACTTGGGATGC |
|  | Arg1 | Forward | TGGCTTGCGAGACGTAGAC |
|  |  | Reverse | GCTCAGGTGAATCGGCCTTTT |
|  | IL-1β | Forward | TTCAGGCAGGCAGTATCACTC |
|  |  | Reverse | GAAGGTCCACGGGAAAGACAC |
|  | β-actin | Forward | CTGAGAGGGAAATCGTGCGT |
|  |  | Reverse | CCACAGGATTCCATACCCAAGA |
